# Heme oxygenase-1 prevents smoke induced B-cell infiltrates: *a role for regulatory T cells*?

**DOI:** 10.1186/1465-9921-9-17

**Published:** 2008-02-06

**Authors:** Corry-Anke Brandsma, Machteld N Hylkema, Barry WA van der Strate, Dirk-Jan Slebos, Marjan A Luinge, Marie Geerlings, Wim Timens, Dirkje S Postma, Huib AM Kerstjens

**Affiliations:** 1 Department of Pulmonary Diseases, University Medical Center Groningen, University of Groningen, P.O. Box 30.001, 9700 RB, Groningen, The Netherlands; 2 Department of Pathology, University Medical Center Groningen, University of Groningen, P.O. Box 30.001, 9700 RB, Groningen, The Netherlands

## Abstract

**Background:**

Smoking is the most important cause for the development of COPD. Since not all smokers develop COPD, it is obvious that other factors must be involved in disease development. We hypothesize that heme oxygenase-1 (HO-1), a protective enzyme against oxidative stress and inflammation, is insufficiently upregulated in COPD.

The effects of HO-1 modulation on cigarette smoke induced inflammation and emphysema were tested in a smoking mouse model.

**Methods:**

Mice were either exposed or sham exposed to cigarette smoke exposure for 20 weeks. Cobalt protoporphyrin or tin protoporphyrin was injected during this period to induce or inhibit HO-1 activity, respectively. Afterwards, emphysema development, levels of inflammatory cells and cytokines, and the presence of B-cell infiltrates in lung tissue were analyzed.

**Results:**

Smoke exposure induced emphysema and increased the numbers of inflammatory cells and numbers of B-cell infiltrates, as well as the levels of inflammatory cytokines in lung tissue. HO-1 modulation had no effects on smoke induced emphysema development, or the increases in neutrophils and macrophages and inflammatory cytokines. Interestingly, HO-1 induction prevented the development of smoke induced B-cell infiltrates and increased the levels of CD4^+^CD25^+ ^T cells and Foxp3 positive cells in the lungs. Additionally, the CD4^+^CD25^+ ^T cells correlated positively with the number of Foxp3 positive cells in lung tissue, indicating that these cells were regulatory T cells.

**Conclusion:**

These results support the concept that HO-1 expression influences regulatory T cells and indicates that this mechanism is involved in the suppression of smoke induced B-cell infiltrates. The translation of this interaction to human COPD should now be pursued.

## Background

Chronic obstructive pulmonary disease (COPD) is a major global health problem with increasing morbidity and mortality. Smoking is widely accepted as the most important cause for development of the disease, still 'only' 15–20% of the smoking population eventually develops COPD [1]. COPD is characterized by a chronic inflammatory process, which ultimately leads to airway obstruction and emphysema. The important role of neutrophils, macrophages and cytotoxic T cells in its development is well established [2], yet the role of CD4 T cells and B cells has only recently re-attracted attention. We and others have found oligoclonal T- and B cells in the lungs of COPD patients suggesting an antigen driven immune response [3,4]. These T-and B cells are aggregated in lymphoid infiltrates. Similar infiltrates have been shown in the lungs of mice chronically exposed to cigarette smoke [3]. We hypothesize that these lymphoid infiltrates contribute to the development and/or persistence of the inflammatory response in COPD [3].

Since not all patients with COPD have actively smoked, cigarette smoke cannot be the sole contributing factor in COPD development. Other factors involved are genetic factors, such as α1 anti-trypsin deficiency, and environmental factors, such as air pollution. Another intriguing factor that may play a role in COPD development is the 'protective' enzyme heme oxygenase-1 (HO-1). HO-1 is the rate limiting enzyme involved in the breakdown of heme to equimolar amounts of biliverdin, free iron and carbon monoxide (CO). HO-1 is rapidly upregulated with oxidative stress and has potent anti-inflammatory, anti-apoptotic and anti-proliferative effects [5-7]. The anti-inflammatory and cytoprotective effects of HO-1 are mediated by its products, of which in particular CO [8-10]. Notwithstanding this knowledge, the exact mechanisms behind the protective effects of HO-1 are still poorly understood.

Interestingly, a reduced HO-1 expression in macrophages in lung tissue and bronchoalveolar lavage (BAL) in patients with COPD has been shown [11,12]. In some people this may be due to a genetic polymorphism in the HO-1 promoter gene, which causes a lower HO-1 inducibility by reactive oxygen species (ROS) [13]. Additionally, adenoviral mediated HO-1 overexpression in the lung suppresses porcine pancreatic elastase induced emphysema development in mice [14], again suggesting involvement of HO-1 in emphysema development.

Our general hypothesis is that if HO-1 is insufficiently upregulated, this contributes to a higher susceptibility to noxious effects of cigarette smoke and subsequent development of COPD. We tested whether HO-1 modulation in our smoking mouse model [3] influences the development of cigarette smoke induced emphysema and lung inflammation, in particular with respect to lymphoid infiltrates. We hypothesized that HO-1 induction attenuates cigarette smoke induced emphysema and inflammation and conversely HO-1 inhibition worsens the noxious effects of cigarette smoke.

This study showed that long term HO-1 upregulation prevented the development of cigarette smoke induced B-cell infiltrates, while it had no effect on smoke induced emphysema and increase in inflammatory cells and cytokines. Increased numbers of CD4^+^CD25^+ ^Tregs could be an explanation for the reduced presence of these B-cell infiltrates.

## Methods

### Study design

Female A/J mice were divided into six groups (n = 11 per group); 1. Phosphate buffered saline (PBS) + smoke, 2. Cobalt protoporphyrin (CoPP) + smoke, 3. Tin protoporphyrin (SnPP) + smoke, 4. PBS + sham smoke, 5. CoPP + sham smoke, 6. SnPP + sham smoke. During 20 weeks the mice were subjected to protoporphyrin (or PBS) treatment and smoke (or sham smoke) exposure. After 20 weeks the mice were sacrificed, the trachea was cannulated, the right lung was ligated, and lung lobes were either snap-frozen and stored at -80°C (n = 7) or freshly used for flow cytometry analysis (n = 7). The left lung was inflated, and fixed for 24 h with formalin with a constant pressure of 25 cm H_2_O (n = 8).

Experiments were approved by the local committee on animal experimentation.

### Smoke exposure

Mice were exposed to 24 puffs of cigarette smoke from two 2R1 reference cigarettes (University of Kentucky) two times per day, for 5 days a week during 20 weeks, as described previously [3].

### Protoporphyrin treatment

CoPP and SnPP (Frontier Scientific, Logan, USA) were dissolved in 1 M NaOH, diluted to the proper concentration with PBS and adjusted to pH 7.3–7.5 with HCl. The mice received a subcutaneous injection with CoPP (25 μM/kg = 16.4 mg/kg) every two weeks, or with SnPP (10 μM/kg = 7.5 mg/kg), or PBS weekly. These concentrations and dosing regimens were based on a pilot, in which different protoporphyrin concentrations were tested for a maximum period of two weeks.

### Morphometrical evaluation of emphysema

Alveolar airspace enlargement was assessed by mean linear intercept (Lmi) by two independent individuals in a blinded manner, as described previously [3,15].

### Cytokines

Frozen lung tissue was homogenized in 50 mM Tris-HCl buffer, containing 150 mM NaCl, and 0.002% Tween-20 (pH 7.5) and centrifuged at 12000 g for 10 min to remove any insoluble material. Concentrations of TNF-α, IL-1α, IL-1β, IL-6, KC (mouse IL-8) and MCP-1 (monocyte chemoattractant protein-1) in supernatants were measured with a multiplex ELISA system (Lincoplex Systems, St Charles, MO, USA).

### Flow cytometry

Single-cell leukocyte suspensions were obtained from lungs for flow cytometric analysis as described previously [16]. Numbers of CD4^+^CD25^+ ^T cells and neutrophils were calculated based on the label combinations: CD3-APC, CD4-PE, CD25-FITC and Gr1-APC. All antibodies were obtained from Pharmingen (San Diego, USA).

### Histology

HO-1 expression was demonstrated with the rabbit polyclonal antibody anti-HO-1 (Stressgen, Victoria, Canada). Macrophage numbers were identified with an anti-Mac3 antibody (Pharmingen) and were quantified by morphometric analysis using Leica Qwin image analysis software (Leica Microsystems BV, Rijswijk, the Netherlands). With this computerized method the total Mac3 positive stained surface area was measured and divided by the total surface area lung tissue, and expressed as volume percentages [16]. B-cell infiltrates were detected with an anti-B220 antibody (Pharmingen). The total surface of the B220 positive infiltrates (clusters of at least 10 cells) was quantified by morphometric analysis and divided by the total surface area lung tissue, and expressed as volume percentages. Forkhead transcription factor 3 (Foxp3) expression, a marker for regulatory T cells, was detected in 4 μm sections of frozen lung tissue by staining with a monoclonal anti-Foxp3 antibody (Alexis, Breda, the Netherlands). The total number of Foxp3 positive cells was counted at 25× magnification and expressed per surface area lung tissue determined by morphometric analysis. A fluorescent double staining with hamster anti mouse-CD3 (Pharmingen), followed by mouse anti hamster FITC-labeled (eBioscience) and rat anti mouse-Foxp3 (Alexis) followed by biotin conjugated goat anti rat (SBA, Birmingen, USA) and Strep-APC (Pharmingen) was performed on 4 μm frozen sections of spleen and lung tissue to confirm that Foxp3 positive cells were T cells.

### Western blot analysis

HO-1 protein expression was measured with western blot in whole lung homogenate (see cytokine analysis). The proteins were separated for molecular weight and blotted on a nitrocellulose membrane. The membrane was blocked overnight in 5% skim milk and incubated with rabbit-anti-HO-1 (Stressgen) followed by a peroxidase labeled goat-anti-rabbit antibody (DakoCytomation, Heverlee, Belgium). For protein loading control the membrane was stripped using a 25 mM Glycine-HCl buffer containing 1% SDS (pH:2) and stained for β-actin (loading control, Abcam, Cambridge, UK) followed by a peroxidase labeled goat-anti-rabbit antibody (DakoCytomation). The bands of interest were visualized using enhanced chemiluminescence according to standard methods.

### Statistics

A multiple linear regression model was used to establish importance of smoke exposure and protoporphyrin treatment and their possible interactions [17]. First, the model was tested with the effects of smoking, CoPP treatment, SnPP treatment, together with the interactions between smoking and CoPP, and smoking and SnPP. When the interactions were not significant the model was tested again without the interaction terms. Afterwards the normal distribution of the residuals was analyzed and when needed the data were log or 1/x-transformed to normalize distributions. A significant interaction signifies that the effect of the combination is different (larger or smaller) than the addition of the separate effects of the exposures. Mann Whitney U tests were used for post-hoc analysis to test whether significant effects of CoPP and SnPP treatment were present only in smokers or sham smokers or in both groups. CD4^+^CD25^+ ^T cells and Foxp3 positive cells were evaluated with the Spearman correlation. A value of p < 0.05 was considered significant.

## Results

### Protoporphyrin treatment and smoking upregulate HO-1 expression

**Figure 1:**
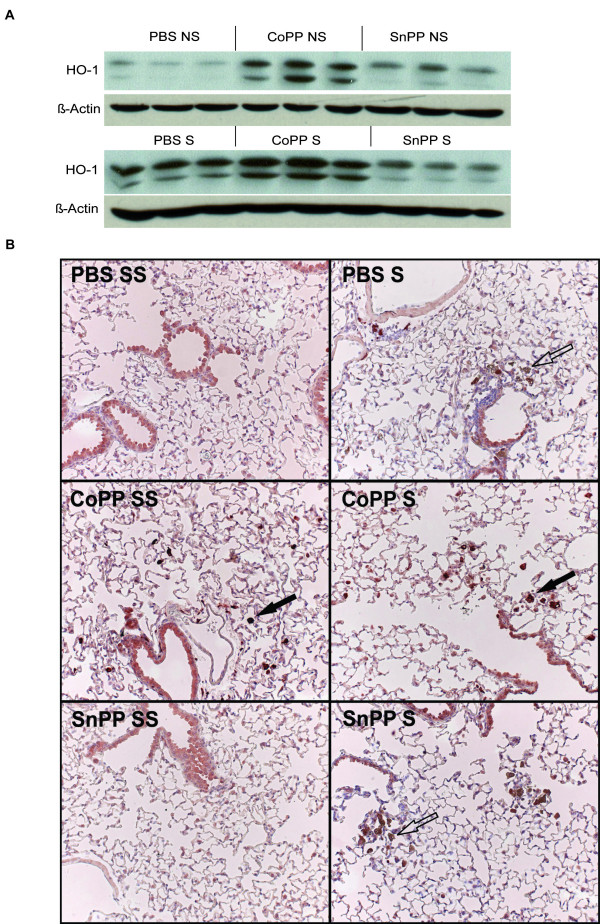
**HO-1 protein expression**. **A: **Protein bands for HO-1 (above band) and β-actin (loading control) detected by western blot analysis after long term smoke exposure and protoporphyrin treatment. Three animals per group are shown. **B: **A representative picture of the HO-1 expression (dark red) in lung tissue is shown for each group (25×). Particularly, alveolar macrophages (indicated with a closed arrow) show an increased HO-1 expression after CoPP treatment. The epithelium stains faintly in all groups and no differences were observed between the groups. The brown cells (indicated with an open arrow) are pigmented macrophages, a result of the smoke exposure. S: smoke, SS: Sham smoke. Mice were divided into 6 groups; 1. Phosphate buffered saline (PBS) + smoke, 2. Cobalt protoporphyrin (CoPP) + smoke, 3. Tin protoporphyrin (SnPP) + smoke, 4. PBS + sham smoke, 5. CoPP + sham smoke, 6. SnPP + sham smoke.

CoPP resulted in a clear upregulation of HO-1 protein expression in the lung, particularly in alveolar macrophages (Figure 1). Smoking also resulted in an increased HO-1 protein expression, leading to highest levels of HO-1 in smoke-exposed mice that also received CoPP. SnPP resulted in a small increase in HO-1 expression, which was not affected by smoking.

### No effects of HO-1 modulation on smoke induced emphysema development

**Figure 2:**
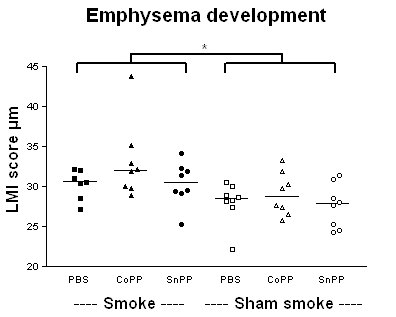
**Emphysema development**. Mean linear intercept (LMI) after long term smoke exposure and protoporphyrin treatment. Smoke groups are represented by closed symbols and sham smoke groups by open symbols. * indicates a significant effect of smoke exposure (p < 0.05). There were no interactions and no effects of CoPP or SnPP treatment.

Smoking induced emphysema after 5.5 months smoke exposure, expressed as a significant increase in mean linear intercept (Figure 2, p < 0.01). There were no effects of both protoporphyrins on the mean linear intercept.

### Smoking increases the levels of inflammatory cytokines in lung tissue

**Figure 3:**
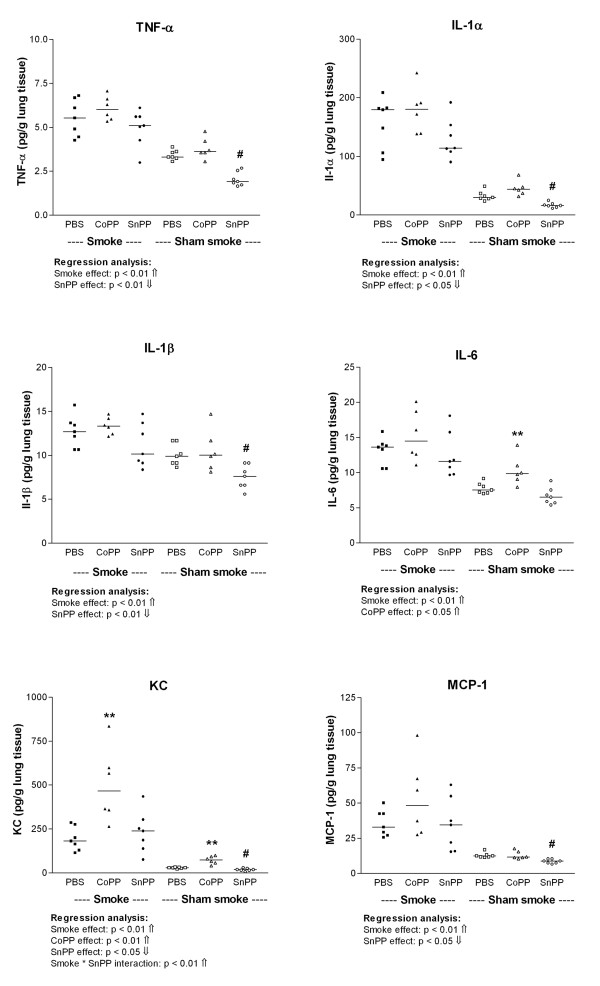
**Inflammatory cytokines in lung homogenate**. TNF-α, IL-1α, IL-1β, IL-6, KC and MCP-1 levels expressed as pg/g lung homogenate after long term smoke exposure and protoporphyrin treatment. Smoke groups are represented by closed symbols and sham smoke groups by open symbols. The significant results of the regression analysis are depicted beneath the figures. ^# ^indicates a significant effect of SnPP treatment (SnPP vs. PBS, post-hoc analysis), and ** indicates a significant effect of CoPP treatment (CoPP vs. PBS, post-hoc analysis) (p < 0.05).

Smoking significantly increased the levels of the pro inflammatory cytokines TNF-α, IL-1α, IL-1β, IL-6, KC, and MCP-1 in lung homogenate (Figure 3, p < 0.01). CoPP increased the levels of IL-6 and KC. Levels of IL-6 were significantly increased after CoPP only in the sham smokers (PBS sham smoke vs. CoPP sham smoke p < 0.05). KC levels were increased after CoPP in both smokers and sham smokers (PBS sham smoke vs. CoPP sham smoke and PBS smoke vs. CoPP smoke p < 0.01). In contrast, SnPP reduced the levels of TNF-α, IL-1α, IL-1β, KC and MCP-1. Levels of these cytokines were significantly decreased after SnPP in the sham smokers only (PBS sham smoke vs. SnPP sham smoke p < 0.05). Additionally, there was a positive interaction between SnPP and smoking for KC (p < 0.01) leading to higher KC levels in the SnPP smokers compared to the SnPP sham smokers.

### Smoking increases neutrophils and macrophages in lung tissue

**Figure 4:**
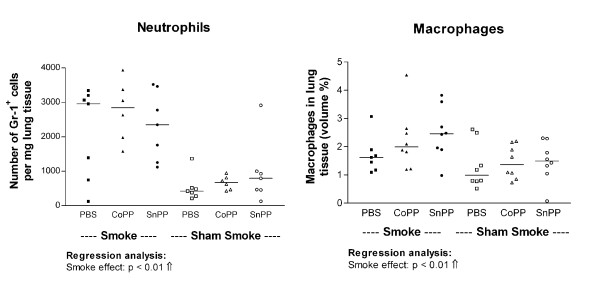
**Inflammatory cells in lung tissue**. Neutrophils and macrophages expressed in lung tissue after long term smoke exposure and protoporphyrin treatment. Neutrophils are expressed as numbers per mg lung homogenate. Macrophages are expressed as volume percentages. Smoke groups are represented by closed symbols and sham smoke groups by open symbols. The significant results of the regression analysis are depicted beneath the figures.

Smoking increased the numbers of neutrophils and macrophages in the lung (Figure 4, p < 0.01). There were no effects of both protoporphyrins on the numbers of neutrophils and macrophages.

### CoPP treatment prevents cigarette smoke induced B-cell infiltrates

**Figure 5:**
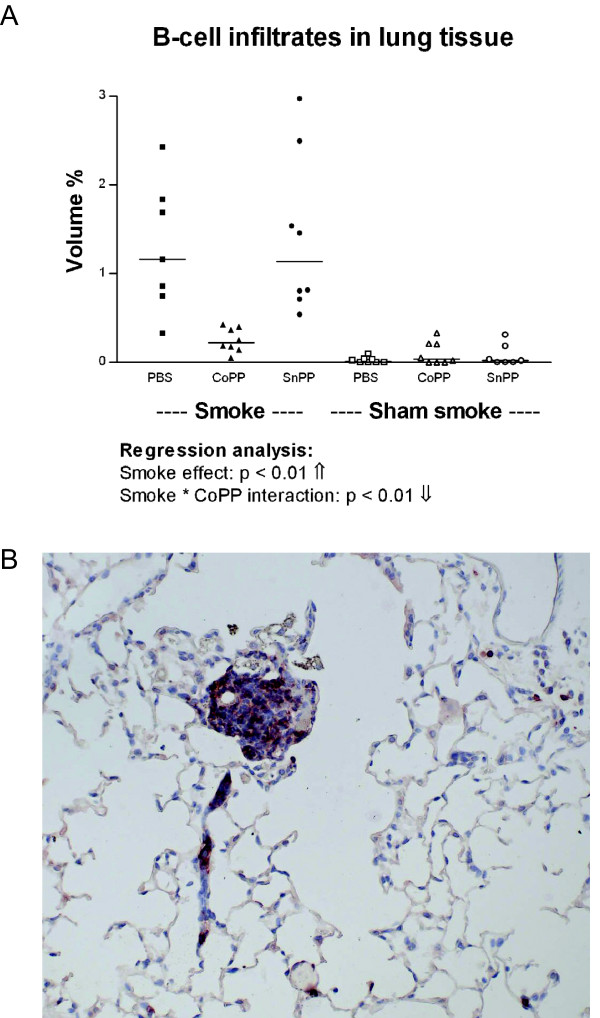
**B-cell infiltrates in lung tissue**. **A: **Volume percentage of B220 positive infiltrates after long term smoke exposure and protoporphyrin treatment. Smoke groups are represented by closed symbols and sham smoke groups by open symbols. The significant results of the regression analysis are depicted beneath the figure. **B: **Example of a B-cell infiltrate positive for B220 (red) present in lung tissue (50×).

Smoking significantly increased the number of B-cell infiltrates in lung tissue (Figure 5, p < 0.01). In addition, there was a significant negative interaction between smoking and CoPP (p < 0.01) signifying that the smoke induced increase in B-cell infiltrates was reduced in the smoke-exposed mice that also received CoPP (CoPP smokers).

### Reduced number of B-cell infiltrates in CoPP smokers is accompanied by increased numbers of CD4^+^CD25^+ ^T cells in lung homogenate

**Figure 6:**
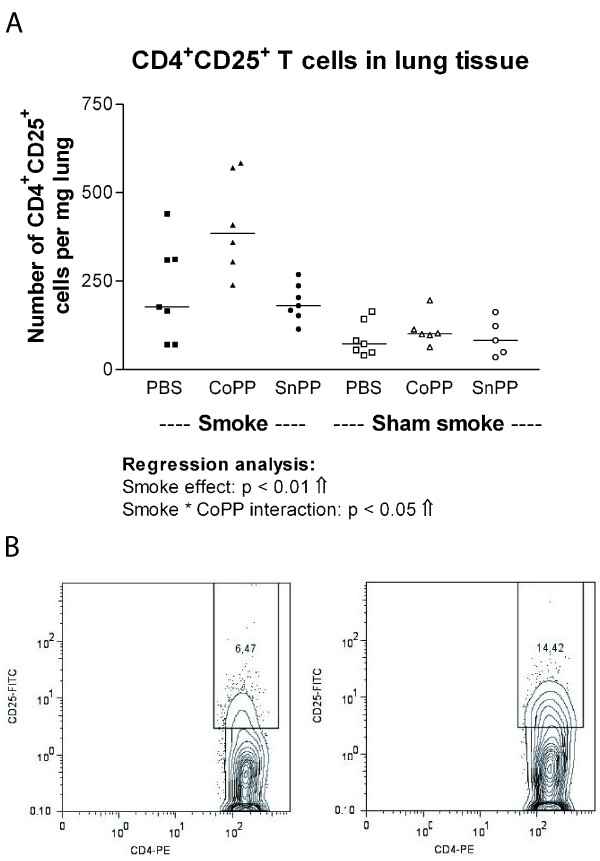
**CD4^+ ^CD25^+ ^T cells in lung tissue**. **A: **CD4^+^CD25^+ ^T cells expressed as numbers per mg lung tissue after long term smoke exposure and protoporphyrin treatment. Smoke groups are represented by closed symbols and sham smoke groups by open symbols. The significant results of the regression analysis are depicted beneath the figure. **B: **Example of the flow cytometry analysis: The percentage of CD25 positive cells within the CD4^+ ^T cell population is shown for a PBS (left) and a CoPP (right) smoking mouse.

Smoking significantly increased the numbers of CD4^+^CD25^+ ^T cells in lung homogenate (Figure 6A, p < 0.01). Additionally, there was a significant positive interaction between smoking and CoPP treatment for the numbers of CD4^+^CD25^+ ^T cells (p < 0.05), signifying that the increase of smoking was larger in combination with CoPP, resulting in the highest numbers of CD4^+^CD25^+ ^T cells in the CoPP smokers. This higher expression of CD25 in the CD4^+ ^T-cell population of CoPP smoking compared to PBS smoking mice is illustrated in Figure 6B.

### The increase in CD4^+^CD25^+ ^T cells represents an increase in regulatory T cells

**Figure 7:**
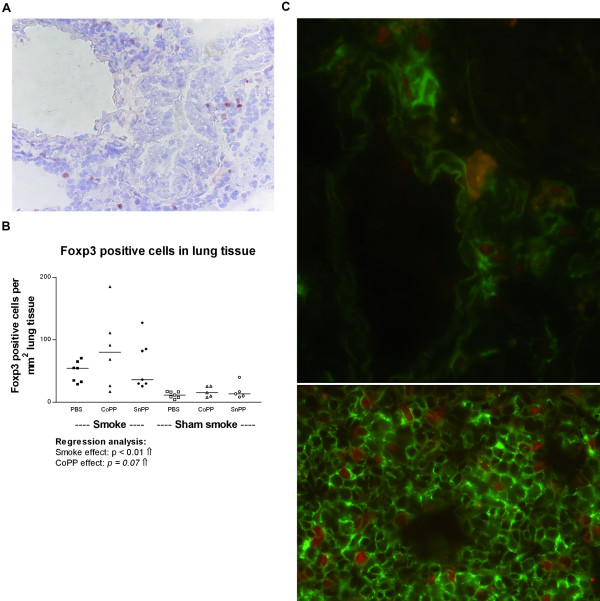
**Foxp3 positive cells in lung tissue**. **A: **Foxp3 positive cells (red nuclear staining) present in lung tissue (100×). **B: **Foxp3 positive cells expressed as total numbers per mm^2 ^lung tissue after long term smoke exposure and protoporphyrin treatment. Smoke groups are represented by closed symbols and sham smoke groups by open symbols. The significant results of the regression analysis are depicted beneath the figure, as well as a trend for an effect of CoPP treatment. **C: **Fluorescent double staining for CD3 (green) and Foxp3 (red) in lung (upper panel, 630×) and spleen (lower panel, 400×) showing that Foxp3 specifically stains T cells. The orange cells in the lung are pigmented macrophages, which show auto fluorescence.

To investigate whether the increased number of CD4^+^CD25^+ ^T cells in the CoPP smokers represented an increase in regulatory T cells (Tregs), we stained lung tissue for the Treg specific marker Foxp3 (Figure 7A). Smoking significantly increased the numbers of Foxp3 positive cells in lung tissue (Figure 7B, p < 0.01) with a trend (p = 0.07) for a similar effect of CoPP on the number of Foxp3 positive cells. Double staining for CD3 and Foxp3 in lung and spleen tissue (Figure 7C) showed that Foxp3 positive cells were indeed T cells. Furthermore, the number of Foxp3 positive cells in lung tissue correlated positively with the number of CD4^+^CD25^+ ^T cells in lung homogenate (ρ = 0.7, p < 0.01).

## Discussion

In this study we showed that HO-1 protein upregulation by CoPP treatment reduced the number of cigarette smoke induced B-cell infiltrates in mice. These B-cell infiltrates were similar to the lymphoid follicles found in COPD patients [3] and are suggested to contribute to COPD development. The reduced number of B-cell infiltrates in the CoPP smokers was accompanied by increased numbers of CD4^+^CD25^+^T cells, which most likely are Tregs. In contrast to our hypothesis, HO-1 upregulation had no protective effect on cigarette smoke induced increases in other inflammatory cells and inflammatory cytokines and subsequent emphysema development. Additionally, SnPP treatment did not aggravate smoke induced damage.

We succeeded in long term HO-1 protein upregulation in our smoking mouse model and to our knowledge this is the first study using an intervention that leads to long term HO-1 protein upregulation *in vivo*. Given the importance of macrophages and epithelium in the production of inflammatory mediators after an inflammatory or oxidative stimulus, these cells were carefully evaluated for their HO-1 expression. In both *in vitro *and *in vivo *studies oxidative stress and cigarette smoke have been shown to induce HO-1 expression in pulmonary epithelial cells and alveolar macrophages [18-21]. Furthermore, HO-1 overexpression in epithelial cells is protective against oxidative stress [22]. Indeed, our study showed an increased HO-1 expression after both cigarette smoke exposure and CoPP treatment. This increased HO-1 expression was highest in CoPP treated smoking mice and particularly seen in alveolar macrophages yet not in epithelial cells. Unfortunately, HO-1 upregulation provided no protective effects against the smoke induced increases in inflammatory cells and cytokines, nor did it protect against smoke induced emphysema. These results do not fit with our hypothesis, but might be explained in several ways. Firstly, the epithelium did not show an increased HO-1 expression after CoPP treatment, which still makes it possible for these cells to respond to cigarette smoke by producing inflammatory mediators. In fact, the majority of the inflammatory cytokines that were increased after smoke exposure in our study can be produced by epithelial cells in response to cigarette smoke [23-26], which supports this option. Secondly, contrary to what we had expected, the levels of IL-6 and KC increased after CoPP treatment in lung tissue, which may suggest some toxicity of the long term dosing of CoPP. Given the fact that the majority of the mice showed irritation of the skin at the injection site after approximately 4 months of CoPP treatment, the CoPP dose indeed might have been too high. It is also conceivable that long term exposure to CoPP, much longer than performed by others, might have other unexpected effects. For future long term experiments it is probably more appropriate to use HO-1 transgenic mice, or use less toxic downstream products of the HO-1 system e.g. CO or bilirubin. Finally, it was not possible to reliably measure HO-activity levels on the frozen material available in this study; a sufficiently sensitive assessment of HO-activity should certainly be included in future experiments.

SnPP treatment resulted in a slightly increased HO-1 protein expression, which was not affected by smoke exposure. In contrast to our hypothesis, SnPP treatment did not aggravate the damaging effects of smoke exposure, but did decrease the levels of several inflammatory cytokines. SnPP is known to inhibit the HO-1 activity, while it increases HO-1 protein expression [27], which supports the increased HO-1 expression after SnPP treatment in our study. For SnPP treatment both inflammatory and anti-inflammatory effects have been described [28-31]. Anti-inflammatory effects were shown while the HO-1 protein level was increased but the HO-1 activity downregulated, suggesting that HO-1 induction by SnPP can have anti-inflammatory and anti-apoptotic effects independently of the HO-1 enzyme activity [30,31].

The most important finding of this study was the protective effect of HO-1 upregulation on the development of cigarette smoke induced B-cell infiltrates, leading to reduced numbers of B-cell infiltrates in CoPP treated smoking mice. The B-cell infiltrates consisted mainly of B cells surrounded by T cells and were comparable to the B-cell follicles found in patients with COPD [3]. B cells in these follicles were found to be oligoclonal in nature [3], suggesting an antigen driven immune response. Whether the inflammatory response in COPD is a true antigen specific response is not fully proven, nor is it clear which antigen(s) may be involved. We consider matrix degradation products, microbial components, and cigarette smoke constituents as possible candidates. We hypothesize that these lymphoid infiltrates contribute to the development and/or persistence of the inflammatory response in COPD. This study showed that reduced numbers of B-cell infiltrates did not prevent smoke induced emphysema development, which suggests that the presence of B-cell infiltrates may not be a mandatory prerequisite for emphysema development in this model. This is compatible with the results of d'Hulst *et al*, showing smoke induced emphysema development in scid mice, lacking functional B- and T-cells [32]. To what extent B cells contribute to the persistence of the inflammatory response in COPD remains unclear. However, since these mouse models of cigarette smoke induced emphysema resemble mild disease, it is also possible that B cells might be more important in severe than in mild disease. This would be supported by the data of Hogg *et al *who found B-cells especially in GOLD stage 3 and 4 [33].

In this study we extended our previous observations on B-cell infiltrates [3] by the intriguing finding that the reduced number of B-cell infiltrates was accompanied by increased numbers of CD4^+^CD25^+ ^T cells in the CoPP smokers. The CD4^+^CD25^+ ^T-cell population consists of a mixture of activated T cells and Tregs. Tregs are important in controlling immunological tolerance and preventing auto-immune reactions by inhibiting T-cell responses [34,35]. Dysfunction of Tregs can lead to auto-immune diseases, allergy, and chronic inflammatory diseases. The currently best described subset of Tregs is that of the naturally occurring Tregs, expressing high levels of CD25 and the transcription factor Foxp3 [34].

In this study, the number of CD4^+^CD25^+ ^T cells correlated positively with the number of Foxp3 positive cells and the highest numbers of Foxp3 positive cells were present in the CoPP smokers with a trend for an effect of CoPP treatment. Together, this suggests that the increase in CD4^+^CD25^+ ^T cells in the CoPP smokers represents an increase in Tregs.

Interestingly, a direct link between Foxp3 and HO-1 expression and function of Tregs was reported recently; both Foxp3 and HO-1 were shown to be expressed in Tregs and the suppressive effects of Tregs were shown to be mediated by HO-1 expression [36]. Furthermore, in a model of allergic airway inflammation, HO-1 upregulation was shown to increase Treg numbers and their suppressive capacity [37].

Next to their effects on T cells, Tregs can also directly suppress B-cell responses without having to suppress the adjacent T cells [38,39]. This proves that activated T cells are not the only target for Tregs and that Tregs can also be involved in the reduced presence of B-cell infiltrates. Interestingly, chronic cigarette smoke exposure was shown to increase the numbers of Tregs in the airways of healthy smokers and smokers with COPD [40], whereas decreased Treg numbers were found in lung tissue of emphysema patients [41]. Additionally, we found high numbers of Foxp3 positive cells present in and surrounding B cell follicles in the lungs of COPD patients (unpublished results).

Altogether, these findings suggest a role for Tregs in COPD in the smoke induced inflammatory response, and possibly also B-cell follicle formation, and support the idea that the HO-1 protein upregulation affected the Treg population in our model thereby possibly contributing to the observed reduced presence of B-cell infiltrates.

## Conclusion

Long term HO-1 upregulation prevented the development of cigarette smoke induced B-cell infiltrates, while it had no effect on smoke induced emphysema and increase in neutrophils and macrophages and inflammatory cytokines. A possible explanation for this effect of HO-1 upregulation on presence of B-cell infiltrates is the increased presence of CD4^+^CD25^+ ^Tregs. The exact role of these Tregs in the smoke induced inflammatory response has to be elucidated and the translation to human COPD should now be pursued.

## Competing interests

The author(s) declare that they have no competing interests.

## Authors' contributions

CB performed the animal experiments, analyzed the data, performed statistical analysis and drafted the manuscript. MH participated in the study design and data analysis, and helped to draft the manuscript. BS participated in the study design and coordinated and performed the animal experiments. ML and MG carried out the flow cytometric, cytokine and immunohistochemical analyses. WT, DP and DS were involved in the study design and critically reviewed the manuscript. HK participated in the study design, was supervisor of the experiments, and helped with the statistical analyses and writing of the manuscript. All authors read and approved the final manuscript.
